# MUSCULOSKELETAL PAIN CHARACTERISTICS AND OBJECTIVELY MEASURED PHYSICAL ACTIVITY IN OLDER ADULTS

**DOI:** 10.1093/geroni/igad104.1966

**Published:** 2023-12-21

**Authors:** Yurun Cai, Fangyu Liu, Amal Wanigatunga, Eleanor Simonsick, Luigi Ferrucci, Jennifer Schrack

**Affiliations:** University of Pittsburgh, Pittsburgh, Pennsylvania, United States; Johns Hopkins Bloomberg School of Public Health, Baltimore, Maryland, United States; Johns Hopkins Bloomberg School of Public Health, Baltimore, Maryland, United States; National Institute on Aging, National Institutes of Health, Baltimore, Maryland, United States; National Institute on Aging, National Institutes of Health, Baltimore, Maryland, United States; Johns Hopkins Bloomberg School of Public Health, Baltimore, Maryland, United States

## Abstract

Pain is associated with reports of restricted physical activity (PA), yet the associations between multiple musculoskeletal pain characteristics and objectively measured PA quantities and patterns in late life are not well understood. A total of 553 adults (mean age 75.8±8.4 years, 54.4% women) in the Baltimore Longitudinal Study of Aging (BLSA) completed pain assessments and subsequent 7-day wrist-worn accelerometry in the free-living environment between 2015 and 2020. Pain characteristics included pain presence in the shoulders, hands/wrists, low back, hip, knees, and feet, pain laterality in each site (no pain, unilateral pain, and bilateral pain), and pain distribution (no pain, single site pain, and multisite pain). PA metrics were summarized into total daily activity counts (TAC), activity fragmentation, active minutes/day, and diurnal patterns of activity. Linear regression models adjusted for demographics and comorbidities suggested that unilateral knee pain was associated with 184,070 fewer TAC/day (p=0.04) and 36.2 fewer active minutes/day (p=0.03) while hand/wrist pain was associated with 24.8 more active minutes/day compared to those without pain (p=0.04). By time-of-day, participants with hip pain had less activity during the morning (6:00am to 11:59am) while those with unilateral knee pain had fewer activity counts during the afternoon (12:00pm to 5:59pm). Analyses stratified by sex showed that these associations were only significant among women. Our study highlights the importance of assessing pain laterality in addition to traditional pain measures (e.g., pain presence) and suggest that pain interferes with multiple aspects of daily activity. Longitudinal studies are needed to assess the temporality of these findings.

